# Supplementary material for: Enhanced nanochannel translocation and localization of genomic DNA molecules using three-dimensional nanofunnels
Source: Nat Commun. 2017 Oct 9;8:807. doi: 10.1038/s41467-017-00951-4 (PMC5634460; doi:10.1038/s41467-017-00951-4)
Supplement: Supplementary file 1 — Supplementary Information [file 41467_2017_951_MOESM1_ESM.pdf]

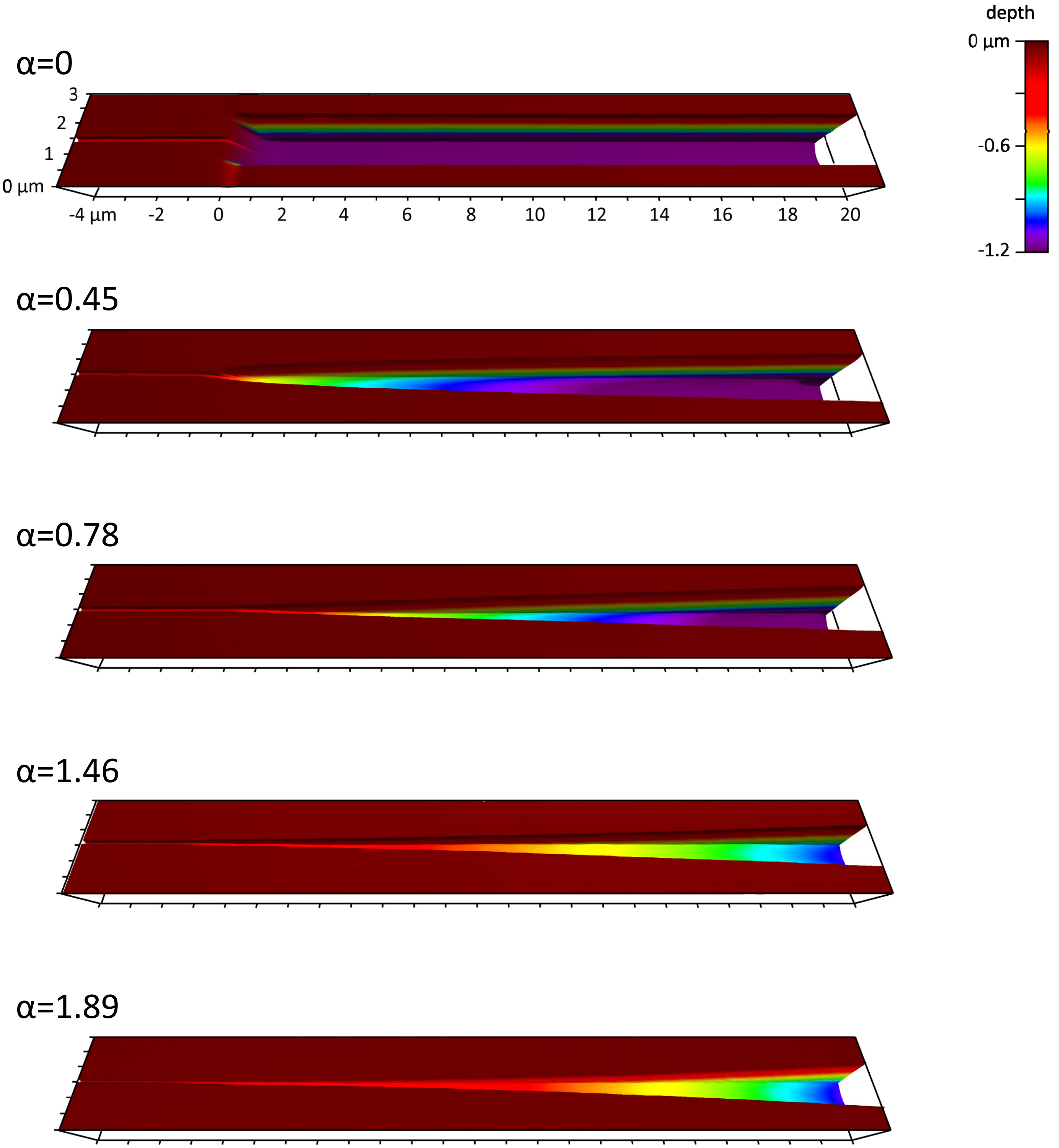


**Supplementary Figure 1 | Atomic force microscopy profiles of three-dimensional nanofunnels.** Each profile includes ~4 µm of the nanochannel and the first ~20 µm of the nanofunnel. The color scale showing the nanofunnel and nanochannel depths facilitates a comparison of how rapidly the depth decreases and the position within the nanofunnels where this decrease primarily occurs.

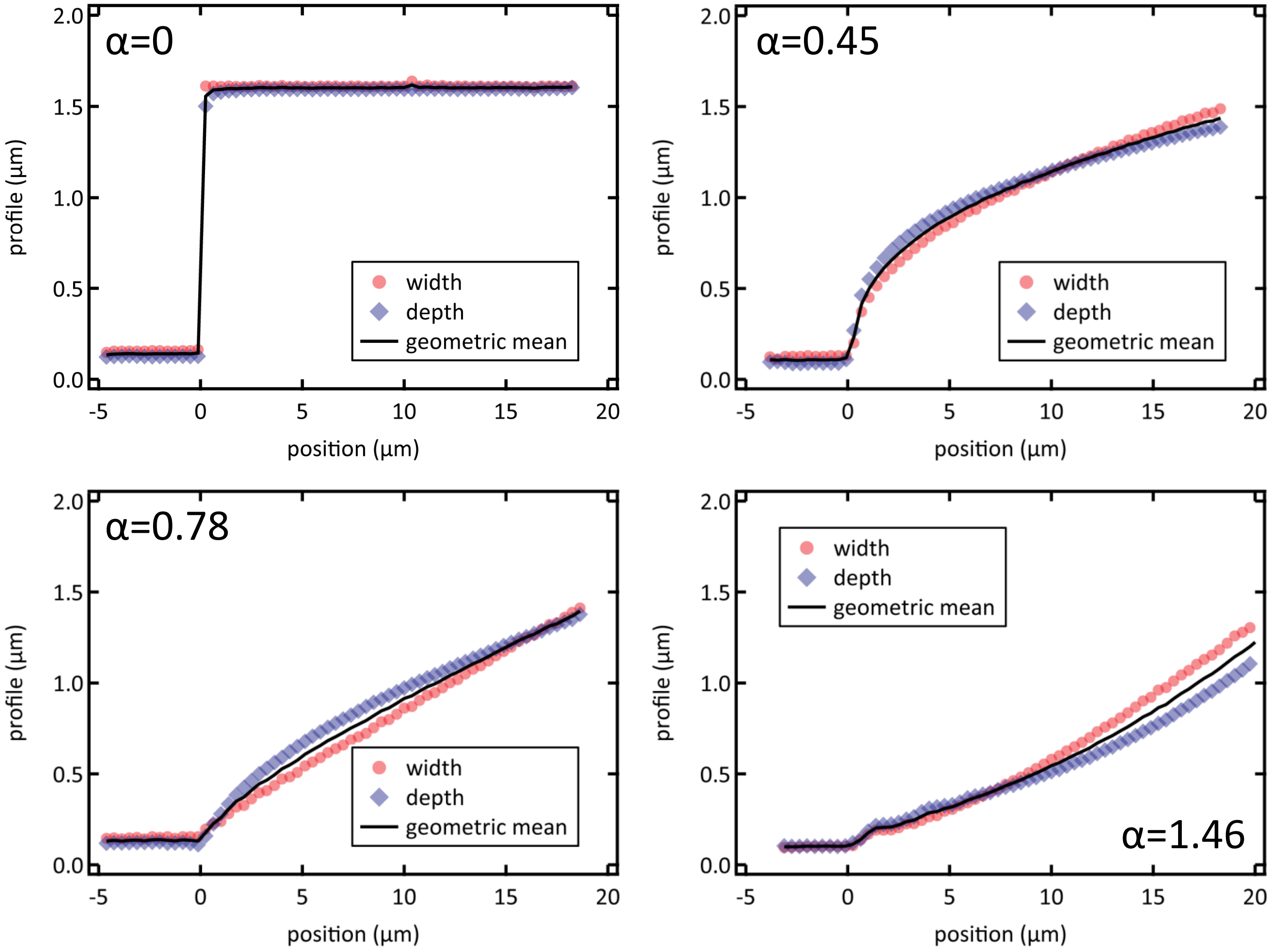


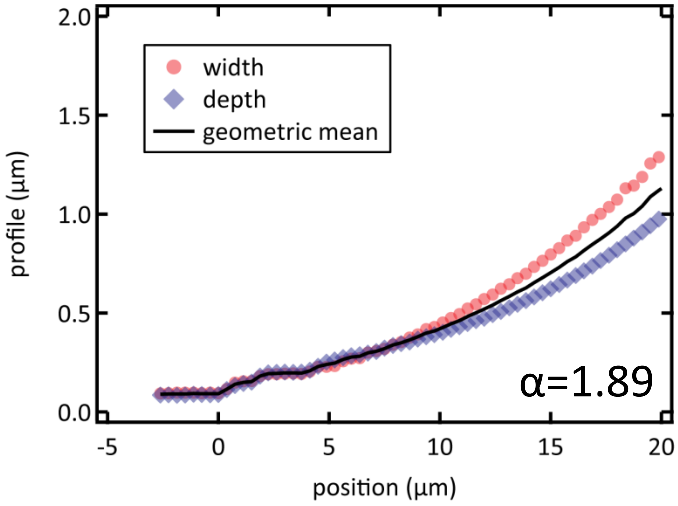


**Supplementary Figure 2 | Width and depth profiles of three-dimensional nanofunnels.** These one-dimensional profiles were extracted from the AFM profiles shown in Supplementary Fig. 1. Each depth profile is measured along a line running down the central longitudinal axis of the nanochannel and nanofunnel. Each width profile is constructed from lines running transverse to the nanochannel and nanofunnel and represents the width of the nanofunnel or nanochannel at the substrate surface. The value of the exponent $\alpha$ that characterized each nanofunnel used in this study was determined by fitting the geometric mean of the nanofunnel width and depth to Eq. 2.

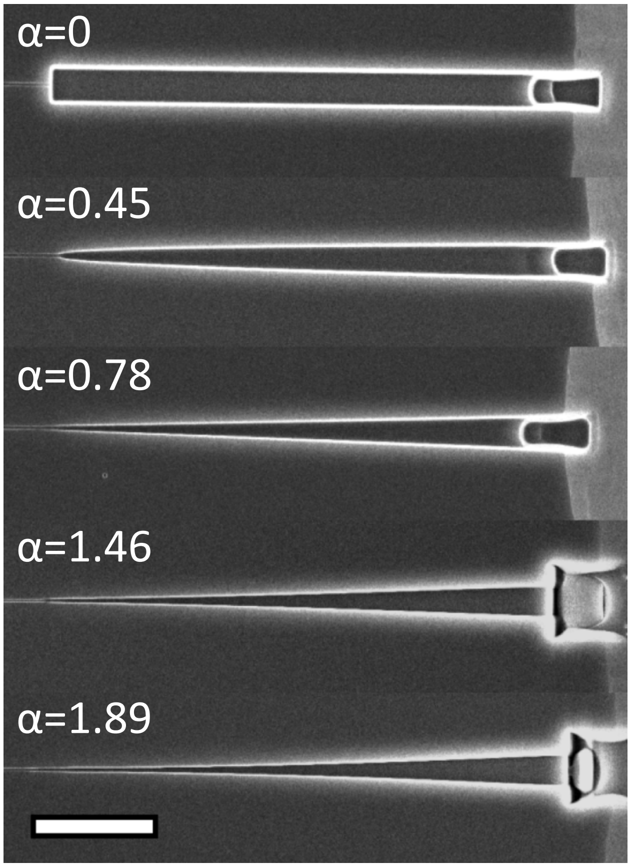


**Supplementary Figure 3 | Top-down scanning electron microscopy images of the nanofunnels used in this study.** Five images are concatenated to facilitate a direct comparison between the five funnels. The values of α that are given describe the power law dependence of depth and width on the position along the nanofunnel. Scale bar is 5 µm.

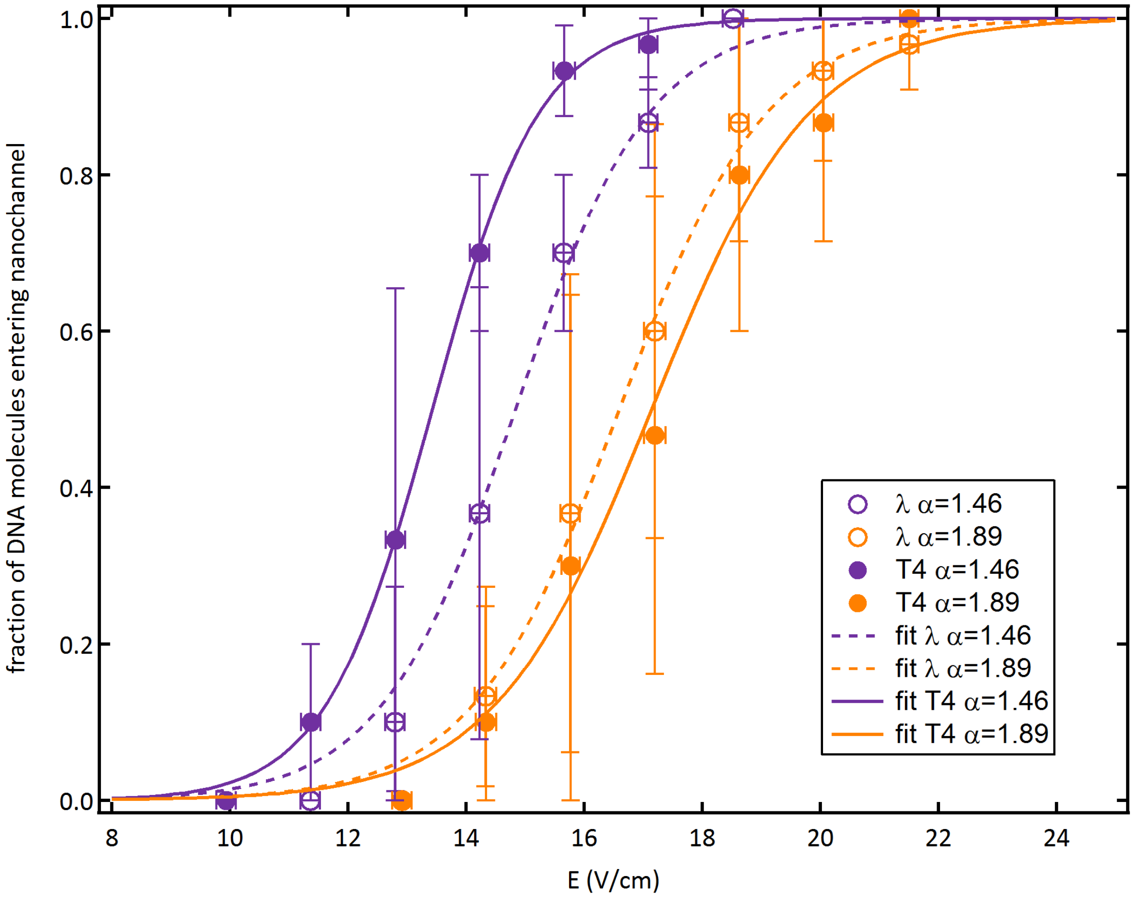


**Supplementary Figure 4 | Threshold measurement results in α=1.46 and α=1.89 nanofunnels.** The error bars are the standard deviations of measurements from 30 individual molecules. The errors are greatest in the middle of the rising portion of the curve as the probability of DNA entry into the nanochannel becomes sensitive to slight variations in DNA starting position and small offset voltages (estimated to be ~1 V/cm) that might originate from pressure driven flow in the system or slight mismatches in the ionic strengths of the solutions on either end of the nanochannel. These data were fit to logistic sigmoid functions. The threshold electric fields are taken from the sigmoidal fits to be the electric fields at which the molecules have a 50% probability of nanochannel entry.

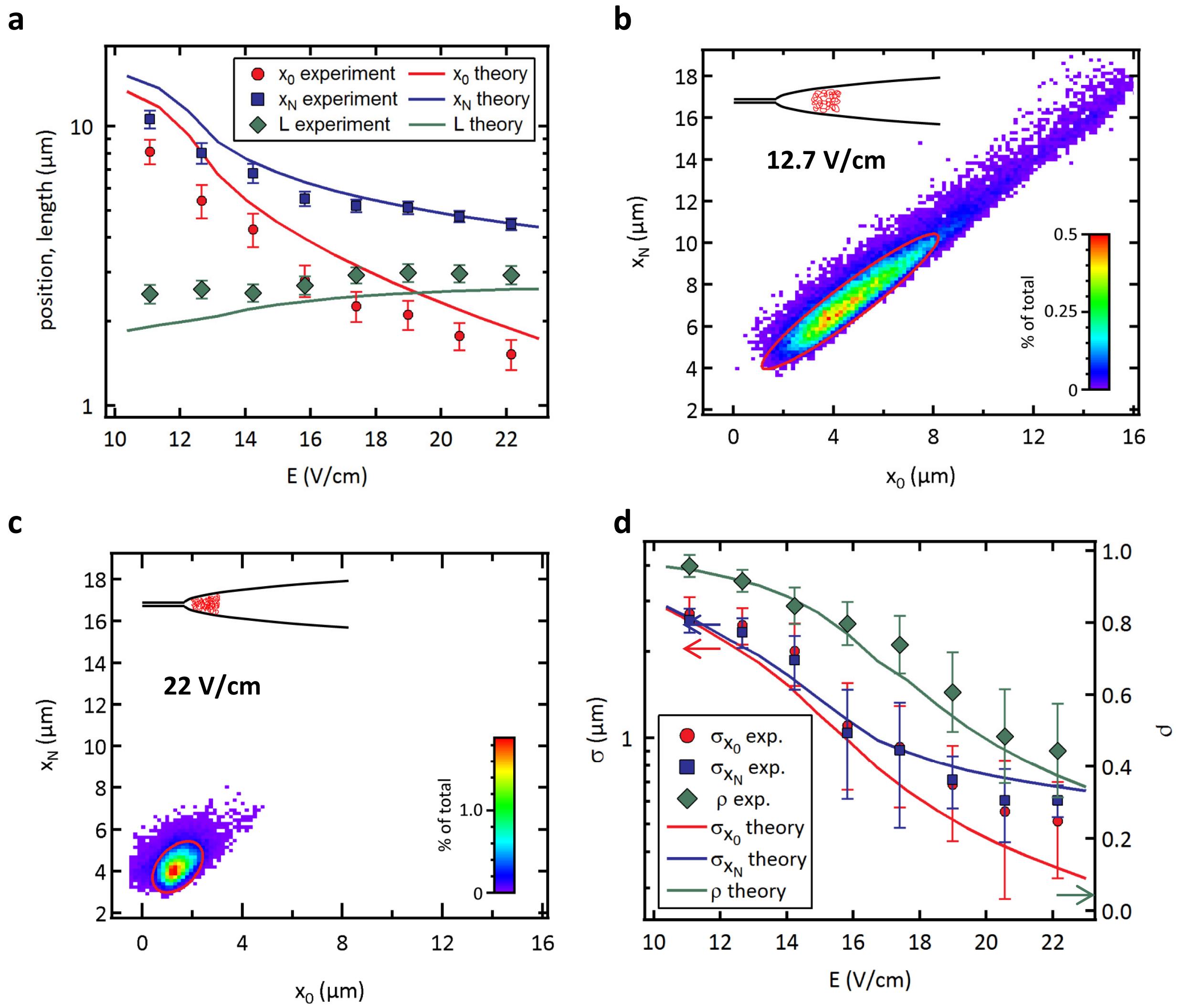


**Supplementary Figure 5 | Field-dependent positions and fluctuations of trapped λ-phage DNA.**
**a,** Experimentally measured mean values of DNA end positions and length (symbols) at each electric field in an α=0.45 nanofunnel compared to the theoretically predicted values (curves). The error bars are the standard deviations of the multiple independent measurements taken over the entire imaging period at a given electric field strength. **b,c,** Filled contour plots showing the probabilities of $x_0$,$x_N$ coordinates measured from each fluorescence image at the low field (12.7 V/cm) and high field (22 V/cm) limits of the stable trapping regime, respectively. The color scales indicate the percentage of measurements corresponding to the various $x_0$,$x_N$ pairs. The insets show schematically the DNA conformations (position, length, packing density) associated with these operating conditions. The red ellipses indicate the best-fit bivariate normal distributions (2σ) to the data. **d,** Comparison of parameters of the bivariate normal distribution fitting analysis of the experimental data (symbols) and theoretical probability distributions (curves). The error bars indicate the 1σ confidence level of the parameters obtained from the bivariate Gaussian fits.

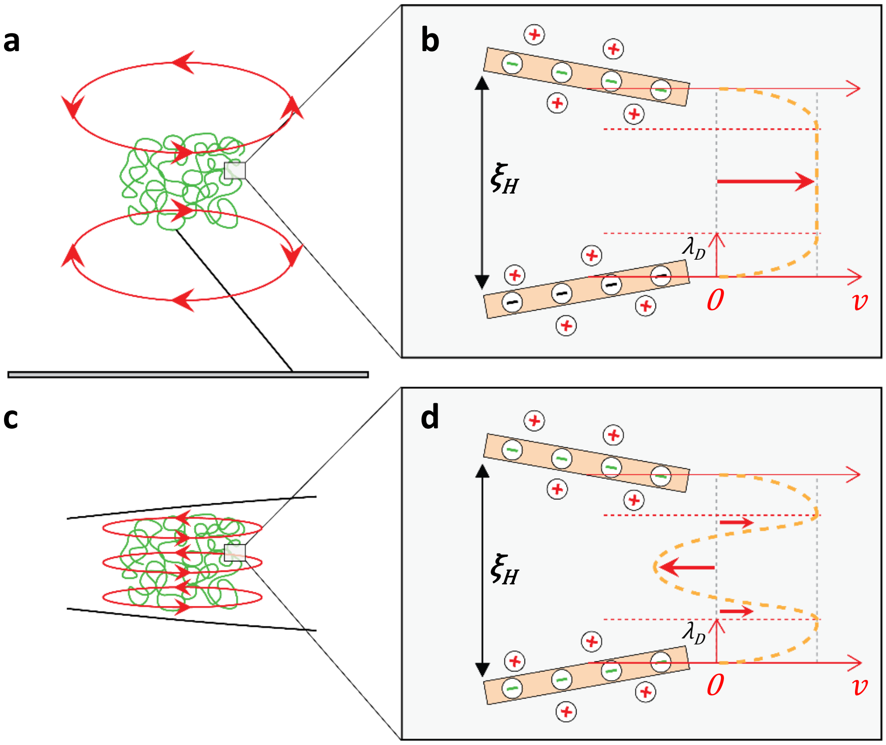


**Supplementary Figure 6 | Electro-osmotically driven fluid flow through a stalled DNA molecule. a,** Flow profile around a DNA molecule stalled in bulk solution. In this hypothetical scenario, the DNA molecule retains its coiled conformation while anchored to a surface that is sufficiently distant that it does not affect the fluid flow through and around the DNA molecule. **b,** Velocity profile in **a** between neighboring DNA segments separated by a mesh size, $\xi_H$. The flow velocity is zero at the surface of the DNA molecule (no-slip condition), reaches a maximum at the Debye length, $\lambda_D$, and is plug-like in the space between DNA segments. **c,** Flow profile around a stalled DNA molecule under confinement in a nanofunnel. The boundary imposed by the nanofunnel walls inhibits flow circulation around the molecule's exterior and flow must circulate through the DNA molecule. **d,** Velocity profile in **c** between neighboring DNA segments in the confined and stalled molecule. The profile is the superposition of electro-osmotic plug-like flow and the pressure-driven backflow through the molecule.

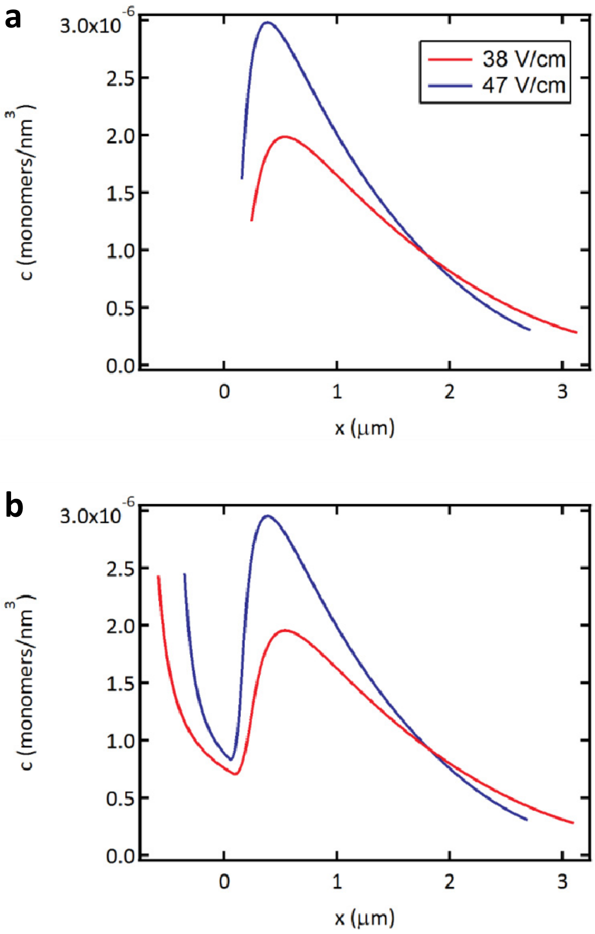


**Supplementary Figure 7 | Concentration profiles for T4-phage DNA in the α=0.45 nanofunnel at two field strengths. a,** Profiles corresponding to the quasi-equilibrium conformation at two different field strengths. **b,** Profiles corresponding to the highest energy ("transition state") conformation at the same field strengths as in **a**. These field strengths coincide with those used in the residence time measurements summarized in Figure 3a of the main text.

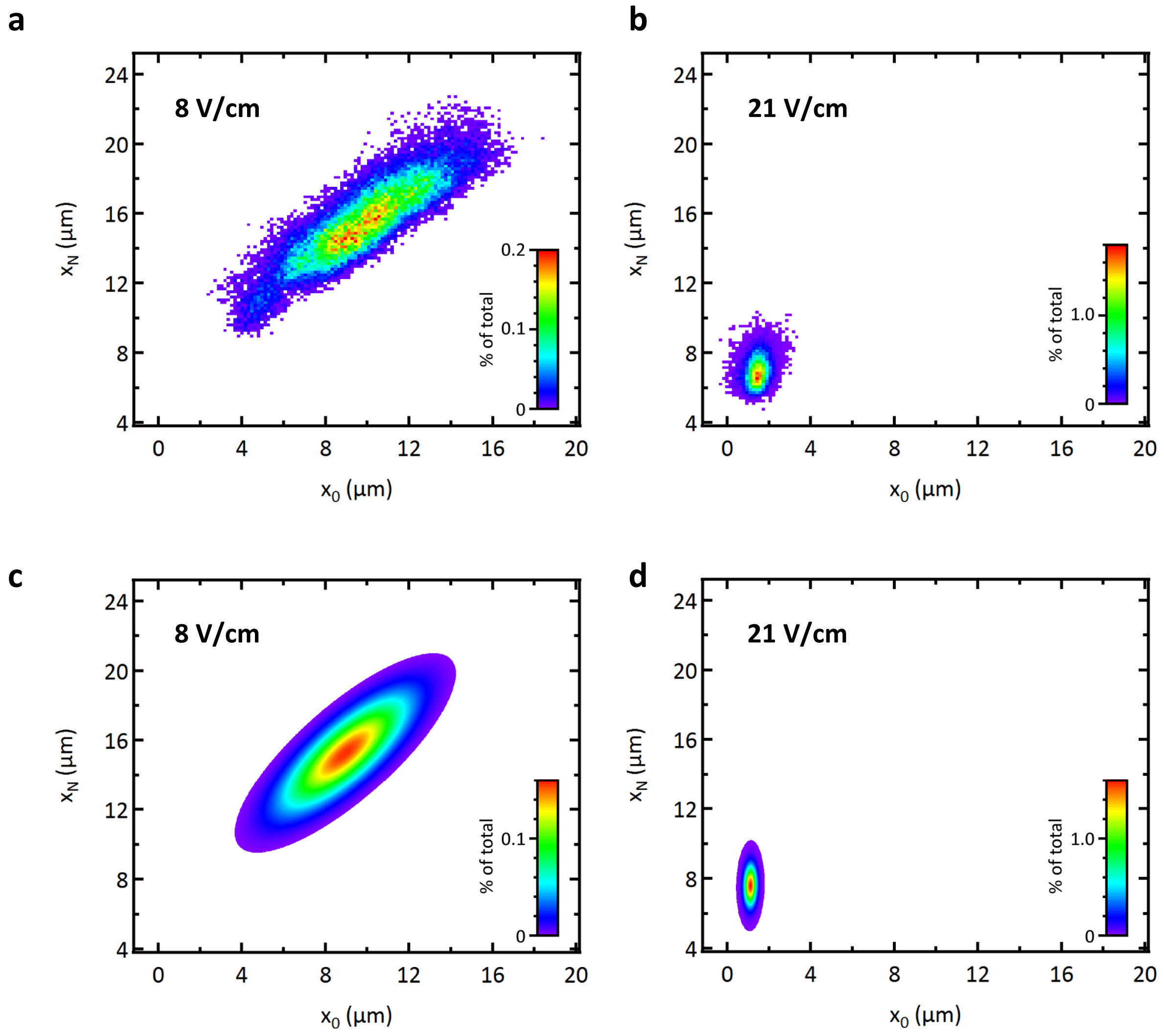


**Supplementary Figure 8 | Comparison of experimental and theoretical $x_0$,$x_N$ distributions for trapping of T4-phage DNA in the α=0.45 nanofunnel. a,b,** Filled contour plots showing the probabilities of $x_0$,$x_N$ values measured from each fluorescence image at the low field (8 V/cm) and high field (21 V/cm) conditions of the stable trapping regime, respectively. **c,d,** Contour plots derived from the probability of displacement from the quasi-equilibrium position at the same low and high field conditions as **a,b**, respectively, as determined by calculating the work necessary for the displacement.

**Supplementary Methods**

Nanofunnel metrology

The dimensions of the FIB-milled three-dimensional nanofunnels were measured using a combination of atomic force microscopy (AFM) and scanning electron microscopy (SEM). AFM was the primary means of profiling the depths and widths of the nanofunnels and nanochannels. Supplementary Fig. 1 shows the three-dimensional renderings of the AFM profiles for each of the nanofunnels. Supplementary Fig. 2 shows depth and width profiles extracted from the AFM scans. SEM imaging was used to measure the length of the nanochannels (which sometimes exceeded the maximum scan region of the AFM), verify the vertical sidewalls of the nanofunnels, and confirm the smoothness of the nanofunnel and nanochannel walls and bottoms. Supplementary Fig. 3 shows top-down SEM images of the various nanofunnels.

Measuring $E_0$ in α=1.46 and α=1.89 nanofunnels

In the class of nanofunnels defined by Equation 2, no stable trapping is observed within a nanofunnel when $\alpha > 1.5$. This is because the depth of the minimum of the effective free energy decreases with increasing alpha due to rapid variation of the diameter along such a funnel. At $\alpha > 1.5$ the well depth becomes smaller than $k_{\mathrm{B}}T$ and thermal fluctuations are sufficient for a DNA molecule to escape the trap. The direction of this escape (towards the narrow end or wide end of the nanofunnel) is highly sensitive to the electric field in the nanofunnel and there is a dramatic difference in the behavior of DNA molecule in a nanofunnel with $\alpha > 1.5$ between the cases of sub-threshold and supra-threshold electric fields. At sub-threshold field strengths, the entropic force dominates and pushes the DNA molecule towards the nanofunnel mouth. Once the molecule exits the nanofunnel, the electric field at the nanofunnel mouth is insufficient to reinitiate entry into the nanofunnel. At supra-threshold field strengths, the electrohydrodynamic and osmotic gradient forces dominate and the molecule is quickly

pulled towards the nanochannel, at which point nanochannel entry is imminent. Intermediate electric field strengths, rather than being characterized by different mean residence times of DNA molecules within the nanofunnel, are characterized by the probability of occurrence of the two outcomes described above. This intermediate range of field strengths was probed by driving a DNA molecule to a set position ($x_0 = 15$ μm) within the nanofunnel, adjusting the nanochannel electric field strength to the test value, and recording whether the molecule entered the nanochannel or diffused out of the nanofunnel mouth. This position was chosen as one that was closest to the mouth of the nanofunnel without allowing the trailing portion of T4-phage DNA molecules to extend out of the nanofunnel and into the microchannel. Entropic recoil initiated by an unconfined portion of the DNA molecules was therefore avoided.[1] Data were collected at increments of 5 mV in the voltage applied across the nanofunnel-nanochannel pairs (corresponding to increments of 1.5 V cm$^{-1}$ in the nanochannel electric field strength). At each field strength 10 molecules were observed and the fraction of molecules that was successfully pulled into the nanochannel was recorded. The measurements were repeated over 3 trials conducted over a span of days at each of the field strengths for each DNA molecule and nanofunnel combination.

Supplementary Fig. 4 shows the measured probability of DNA entry into the nanochannel at various nanochannel electric field strengths for both λ-phage and T4-phage DNA in $\alpha = 1.46$ and $\alpha = 1.89$ nanofunnels. These data were fit to logistic sigmoid functions. The threshold electric fields are taken from the sigmoidal fits to be the electric fields at which the molecules have a 50% probability of nanochannel entry. In the $\alpha = 1.46$ nanofunnel, T4-phage DNA entered the nanochannel more facilely than λ-phage DNA ($E_0 = 13.4 \pm 0.4$ V cm$^{-1}$ and $E_0 = 14.8 \pm 0.5$ V cm$^{-1}$, respectively), continuing the trend seen in nanofunnels with smaller $\alpha$ values. As predicted by theoretical calculations, the threshold electric fields were slightly higher in the case of the $\alpha = 1.89$ nanofunnels. The difference between the T4-phage threshold field strength ($17.2 \pm 0.9$ V cm$^{-1}$) and that of λ-phage ($16.6 \pm 0.6$ V cm$^{-1}$) within the

$\alpha = 1.89$ nanofunnel was statistically indistinguishable. We note that these values should be taken as reasonable estimates when used in a direct comparison with the residence time measurements (as in Fig. 3b, main text), given the fundamentally different methods of measurement. We further note that one of the primary conclusions of this study — that the threshold electric field needed to initiate nanochannel entry is reduced 30-fold — is indicated not only by the $\alpha = 1.46$ and $\alpha = 1.89$ results but also by the residence time measurements conducted in the $\alpha = 0.78$ nanofunnel. That is, the asymptotic behavior occurring at larger values of $\alpha$ (as the strength of the electro-osmotic piston is saturated) is spanned by multiple data sets.

<u>Trapping of λ -phage DNA using Electro-Osmotic Tweezers</u>

The controlled trapping of both λ-phage and T4-phage DNA were experimentally investigated and theoretically modeled. Supplementary Fig. 5 shows the results for a λ-phage DNA molecule trapped in a $\alpha = 0.45$ nanofunnel at various electric field strengths. In Supplementary Fig. 5a, the evidence of DNA compression is present but less dramatic than in the case of the larger T4-phage DNA molecule (Fig. 4a, main text). The molecule's extension length, $L$, continues to increase with an increase in electric field strength but this increase is less than that predicted in the absence of an electric field.[2] Furthermore, the λ-phage DNA molecule exhibits larger fluctuations of the end positions ($\sigma_{x_0}$, $\sigma_{x_N}$) and a greater correlation ($\rho$) between these fluctuations than the T4-phage DNA molecule. This is evident through a qualitative comparison of Supplementary Figs. 5b,c and Figs. 4b,c (main text) and quantitatively in the values plotted in Supplementary Fig. 5d (and Fig. 4d, main text). These findings are consistent with the greater diffusivity of the smaller λ-phage DNA molecule. The values of $\sigma_{x_0}$, $\sigma_{x_N}$, and $\rho$ plotted in Supplementary Fig. 5d are compared to the theoretical predictions of λ -phage DNA's thermal fluctuations (see Supplementary Eqs. 36-45 below).

## Supplementary Discussion

In this Supplementary Discussion, we present a theoretical description of the behavior of a DNA molecule confined in a three-dimensional nanofunnel by the applied electric field. The double-stranded DNA is modeled as a semi-flexible chain having a Kuhn length, $b$, of 100 nm;[3] a geometric backbone diameter, $a$, of 2 nm; and an effective width, $w$, of 6 nm that includes the contribution of the double layer formed by counterions around the backbone.[4] The widths and depths of the nanochannels and nanofunnels used in this study are sufficiently large (i.e., greater than $b$) to allow the random coiling of the DNA molecule. The large aspect ratio b:w of the double-stranded DNA results in relatively weak excluded volume, three-body, and higher-body interactions between Kuhn segments. Solutions of polymers with such large aspect ratio Kuhn segments, called marginal solutions,[5] are qualitatively different from "ordinary" polymer solutions in theta or good solvent, as will be discussed below. At a given electric field strength, the DNA molecule assumes a mean position and conformation that is determined by the local balance of the forces acting upon each of its segments. These are the confinement force, the elastic force, the electrohydrodynamic force, and the osmotic gradient force. The confinement and elastic forces are both entropic in origin and together constitute the entropic force referred to in this Article.

***Confinement force.*** The narrower the funnel cross-section, the more contacts the DNA molecule has with the funnel walls and the greater its confinement free energy. The confinement free energy per unit volume, $F_{\text{conf}}$, is given by

$$F_{\text{conf}} = \begin{cases} k_{\text{B}}Tc(x)\, b^2A^{-1}(x) & \text{if } c(x) < b^2/(vA(x)) \quad (1) \\ k_{\text{B}}Tc^{3/2}(x)\, v^{1/2}bA^{-1/2}(x) & \text{if } c(x) > b^2/(vA(x)) \quad (2) \end{cases}$$

where $c(x)$ is the number density of Kuhn segments at coordinate $x$ along the funnel axis, $A(x)$ is the cross-sectional area at coordinate $x$, and $v$ is the excluded volume parameter ($v \approx b^2 w$).[5-7] The density is normalized to the total number of DNA Kuhn segments:

$$\int_{x_0}^{x_N} c(x)A(x)\,dx = N \tag{3}$$

Supplementary Equations 1 and 2 correspond to regimes of low and high monomer density, respectively. The low monomer density regime is defined by a polymer correlation length, $\xi(x) \approx b/\sqrt{c(x)\,v}$, that is greater than the nanofunnel width and depth, in which case all of the DNA segments in volume $A(x)dx$ feel the effects of confinement.[5,8] The high monomer density regime occurs if the compression of the DNA molecule reduces the correlation length so that it is smaller than the nanofunnel width and depth. In this case only those DNA segments that are within a distance $\xi(x)$ from the nanofunnel walls are perturbed by the walls. DNA segments in the nanofunnel interior (located farther than $\xi(x)$ away from the nanofunnel walls) do not incur an entropic penalty. We found that, for the nanofunnels and DNA molecules investigated in this study, all of the DNA segments were described by the low-density regime and Supplementary Equation 1 is used in the equations below.

The variation of the confinement free energy with the location $x$ of the molecule's segments gives rise to a confinement gradient force that pushes the molecule towards the mouth of the nanofunnel. This confinement gradient force, $f_{\mathrm{conf}}$, expressed per Kuhn monomer, is the derivative of the confinement free energy density:

$$f_{\mathrm{conf}} = -\frac{\partial}{\partial x}\left(\frac{\partial F_{\mathrm{conf}}}{\partial c}\right) \tag{4}$$

The expression in the parentheses can be thought of as the contribution of the confinement to the effective monomeric chemical potential.

***Elastic force.*** The conformational entropy of a macromolecule is also reduced when it is forcibly stretched by pulling on one or both ends of the molecule.[7,9] The general expression for the elastic free energy of a randomly coiled polymer is $k_B T R^2/R_0^2$, where $R$ is the end-to-end distance of the extended molecule and $R_0$ is its unperturbed end-to-end distance.[7] In the case of the nanofunnel-confined DNA molecule, we consider a segment of length $dx$ containing $c(x)A(x)dx$ monomers. The unperturbed mean-square end-to-end distance of each such segment is $b^2 c(x)A(x)dx$. The elastic free energy of the segment is $k_B T(dx)^2/[b^2 c(x)A(x)dx]$. Since the volume occupied by this segment is $A(x)dx$, the elastic free energy per unit volume, $F_{\mathrm{elas}}$, can be written as

$$F_{\mathrm{elas}} = \frac{k_B T}{[bA(x)]^2 c(x)} \tag{5}$$

The elastic force per Kuhn monomer is then

$$f_{\mathrm{elas}} = -\frac{\partial}{\partial x}\left(\frac{\partial F_{\mathrm{elas}}}{\partial c}\right) \tag{6}$$

where the expression in the parentheses is the elastic contribution to the effective monomeric chemical potential. This elastic contribution is significant for the "transition state" conformations where the leading end of the DNA molecule is inserted into the nanochannel and is significantly stretched by the nanochannel electric field. The elastic contribution is negligible for the lowest energy conformations.

***Electrohydrodynamic force.*** The electrostatic force acting on a Kuhn monomer of the DNA molecule is

$$f_{\mathrm{el}} = qE(x) \tag{7}$$

where $q$ is the charge per Kuhn monomer and $E(x)$ is the electrostatic field at coordinate $x$. While each nucleotide pair has a nominal charge of 2*e*, counterion condensation partially shields the backbone charge, reducing the charge per Kuhn monomer to $q = eb/l_B = 143\ e$, where $l_B = 0.7$ nm is the Bjerrum length at which two elementary charges *e* interact with thermal energy $k_B T$.[10] Given the

polarity of the applied electric field (Fig. 1, main text), the electrostatic force pulling on the DNA monomers is directed from the mouth to the narrow end of the nanofunnel.

The DNA backbone is further surrounded by a cloud of uncondensed counterions (cations) localized within the double layer, delimited by the Debye length, $\lambda_{\mathrm{D}}$, of the solution. The electrostatic force acting on the counterions drives them from the narrow end to the wide end of the nanofunnel, in the direction opposite to the force acting on the polyanionic DNA molecule. The migrating cations transmit the electrostatic force through friction to the surrounding fluid, inducing electro-osmotic flow and resulting in a hydrodynamic drag force, $f_{\mathrm{drag}}$, acting from the induced flow onto the DNA. The magnitude of $f_{\mathrm{drag}}$, averaged over the cross-sectional area defined by the nanofunnel shape, depends on the fluid flow profile between DNA strands, which in turn depends on the molecular conformation of the DNA and on the presence of the nanofunnel walls. It is important to note that the DNA molecule acts as a weak electro-osmotic pump, generating a pressure difference across the molecule. Whether this pressure difference is sufficient to draw fluid flow from the nanochannel or results in a backflow through the "pores" (paths between strands) in the DNA molecule depends on the relative hydraulic resistances of the two paths. Based on the dimensions of the micro- and nanoscale channels and the monomer density of a nanofunnel-trapped T4 DNA molecule, we estimate that the backflow through the DNA molecule has a resistance that is two orders of magnitude lower than flow through the nanochannel.[11] Note that in contrast to the case of a DNA molecule stalled in bulk solution, where fluid flow is circulated around the exterior of the DNA molecule, the presence of the nanofunnel walls forces the flow through the DNA molecule (Supplementary Fig. 6)[12,13] The resulting flow profile therefore corresponds to that seen in the case of electro-osmotic flow with a counteracting pressure driven flow.[14]

If there were no force transmitted to the bulk fluid and no pressure gradient created by it, all of the force from the electric field acting on the counterions would be transmitted back to the DNA

backbone by the hydrodynamic flow created by counterions migrating within the double layer. Therefore, the drag force on a stationary DNA molecule in general consists of two parts: the direct drag force, $f_{\mathrm{drag}}^{\mathrm{el}} = -f_{\mathrm{el}}$, compensating the electrostatic force, (Supplementary Eq. 7), and the drag force $f_{\mathrm{drag}}^{u}$ due to electro-osmotic flow through the pores of the molecule and the coincident backflow with combined velocity $u$:

$$f_{\mathrm{drag}}(x) = -qE(x) + f_{\mathrm{drag}}^{u}(x) \tag{8}$$

As noted above, the backflow observed for a DNA molecule filling the cross-section of the nanofunnel proceeds through the pores inside the entire volume of the molecule. The hydrodynamic resistance of the compressed DNA to the flow through it is analogous to the resistance of a porous medium with a pore size on the order of the mesh size, $\xi_{\mathrm{H}}(x)$,[15] which is the average distance between neighboring strands:

$$\xi_{\mathrm{H}}(x) \approx [c(x)b^2]^{-1} \tag{9}$$

varying reciprocally with the monomer number density, $c(x)$. We note that the mesh size (also referred to as the hydrodynamic screening length) is distinct from the correlation length, $\xi(x)$, determining concentration fluctuations and osmotic pressure. $\xi_{\mathrm{H}}(x)$ is a purely geometric distance between neighboring strands of double-stranded DNA and does not depend on the chain thickness or the strength of excluded volume repulsions. For the marginal solvent case, $\xi(x) > \xi_{\mathrm{H}}(x)$ with the ratio $\xi(x)/\xi_{\mathrm{H}}(x) = b^2c^{1/2}w^{-1/2} \gg 1$.

The hydrodynamic friction force acting on chain section with size $\xi_{\mathrm{H}}$ due to flow with velocity $u$ depends on the relative values of this mesh size and the Kuhn length $b$. In the case where $\xi_{\mathrm{H}} \gg b$, corresponding to coil-like chain sections, the Stokes-like friction force on a section of size $\xi_{\mathrm{H}}$ is on the order of $-6\pi\eta u\xi_{\mathrm{H}}$. In the opposite case where $\xi_{\mathrm{H}} \ll b$ the frictional force on the rod-like chain sections is $\approx -2\pi\eta u\xi_{\mathrm{H}}/\ln(\xi_{\mathrm{H}}/a)$. The corresponding friction force on a DNA molecule per Kuhn monomer is

then $-6\pi\eta u b^2/\xi_{\mathrm{H}}$ in the case of coil-like mesh segments and $-2\pi\eta u b/\ln(\xi_{\mathrm{H}}/a)$ for rod-like mesh segments. These two limiting cases can be combined in a cross-over expression for the drag force per Kuhn monomer

$$f_{\mathrm{drag}}^{u} \approx -\frac{2\pi\eta u b}{\ln(\xi_{\mathrm{H}}/a) + \xi_{\mathrm{H}}/(3b)} \tag{10}$$

In general, the flow velocity through the DNA molecule can be due to an external pressure gradient, background electro-osmotic flow within the nanofunnel resulting from surface charges on the device walls, and the electro-osmotic flow induced by counterions associated with the DNA molecule plus the coincident backflow. In our experiments, there is no external pressure gradient, but the negative charge of the fused silica walls of the device results in the electro-osmotic fluid flux $Q$ through it with velocity $u_{\mathrm{eo}}(x) = Q/A(x)$.[16]

By analogy with the electrophoretic velocity of a free-flowing molecule, the DNA counterion-induced velocity is proportional to the product of the electric field $E(x)$ and effective linear charge density $q/b$:

$$u_{\mathrm{bf}} = -\frac{q}{b}\frac{E\lambda_{\mathrm{D}}}{2\pi\eta a} \tag{11}$$

where $\lambda_{\mathrm{D}}$ is the Debye length of the solution. The total drag force per Kuhn monomer is:

$$\begin{aligned} f_{\mathrm{drag}} = f_{\mathrm{drag}}^{\mathrm{el}} + f_{\mathrm{drag}}^{u} &= -qE(x) - \frac{2\pi\eta b(u_{\mathrm{bf}} + u_{\mathrm{eo}})}{\ln[\xi_{\mathrm{H}}(x)/a] + \xi_{\mathrm{H}}(x)/(3b)} \\ &= -qE(x) + \frac{q(\lambda_{\mathrm{D}}/a)E(x) - 2\pi\eta b Q/A(x)}{\ln[\xi_{\mathrm{H}}(x)/a] + \xi_{\mathrm{H}}(x)/(3b)} \end{aligned} \tag{12}$$

In our experiments, the mesh size $\xi_{\mathrm{H}}$ is smaller than $3b\ln[\xi_{\mathrm{H}}(x)/a]$ and we therefore ignore the second term in the denominator in Supplementary Equations 10 and 12. Combining Supplementary Equations 7 and 12 gives the total electrohydrodynamic force, $f_{\mathrm{eh}}$, per Kuhn monomer.

$$f_{\mathrm{eh}} = f_{\mathrm{el}} + f_{\mathrm{drag}} = \frac{q_{\mathrm{red}}(\lambda_{\mathrm{D}}/a)E(x)}{\ln[\xi_{\mathrm{H}}(x)/a]} \tag{13}$$

In Supplementary Equation 13, we account for the electro-osmotic flow due to the surface charges on the walls of the device as a reduction in the effective charge on the DNA molecule:

$$q_{\mathrm{red}} = q - \frac{2\pi\eta ab}{\lambda_{\mathrm{D}}}\frac{Q}{EA_{\mathrm{nc}}} \tag{14}$$

where $E$ is the electric field in the nanochannel with cross-sectional area $A_{\mathrm{nc}}$. This approach can be considered as an extension of the electrohydrodynamic equivalence principle formulated in Reference 12, where in the current work the backflow of solvent is through the molecule — due to the presence of the nanofunnel walls — rather than around the molecule.

Electrophoresis is inherently a non-equilibrium phenomenon but the stalled DNA molecule can be approximated as having a quasi-equilibrium conformation. The impact of the electric field on the DNA conformation and its location along the funnel can then be considered as resulting from an effective potential difference

$$V(x) = \int_{x_0}^{x} \frac{(\lambda_{\mathrm{D}}/a)E(x')}{\ln[\xi_{\mathrm{H}}(x')/a]} dx' \tag{15}$$

and the effective free energy density, $F_{\mathrm{eh}}$, of the electrohydrodynamic forces is

$$F_{\mathrm{eh}} = q_{\mathrm{red}}V(x)c(x) \tag{16}$$

***Osmotic gradient force.*** There is also a free energy contribution from the interactions between segments of the DNA molecule, $F_{\mathrm{int}}$. The interaction free energy density between the segments of a worm-like chain with high-aspect-ratio Kuhn segments can be expressed in the marginal solvent regime as a two-body repulsion term:

$$F_{\mathrm{int}} = \frac{k_{\mathrm{B}}T}{2}\nu c^2(x) \tag{17}$$

Since the concentration of DNA segments varies along the longitudinal axis of the nanofunnel (Supplementary Fig. 7), there is a gradient of the interaction free energy that induces a force pushing chain segments from high to low density regions. This force, $f_{\mathrm{osm}}$, has the characteristics of an osmotic gradient force as it opposes the increase of DNA concentration.

$$f_{\mathrm{osm}} = -\frac{\partial}{\partial x}\left(\frac{\partial F_{\mathrm{int}}}{\partial c}\right) = -k_{\mathrm{B}}Tv\,\frac{dc(x)}{dx} \tag{18}$$

***Total effective free energy.*** Combining the four free energy density terms (Supplementary Equations 1, 5, 16, 17) and integrating their sum over the DNA volume $\int_{x_0}^{x_N} \cdots A(x)dx$ results in the expression for the total effective free energy of the molecule:

$$\frac{F}{k_{\mathrm{B}}T} = P_1 \int_{x_0}^{x_N} b^2 c(x)\,dx + P_2 \int_{x_0}^{x_N} \frac{1}{b^2 A(x)c(x)}\,dx + P_3 \int_{x_0}^{x_N} \frac{v}{2} c^2(x)A(x)\,dx + \int_{x_0}^{x_N} \frac{q_{\mathrm{red}}}{k_{\mathrm{B}}T} c(x)V(x)A(x)\,dx \tag{19}$$

with the additional normalization condition on monomer density (Supplementary Eq. 3). The three numerical coefficients $P_1$, $P_2$, and $P_3$ are of order unity and the potential $V(x)$ is defined in Supplementary Equation 15.

***Effective free energy barrier.*** The effective free energy was evaluated to determine the DNA conformation at the lowest energy, $F_{\mathrm{min}}$ – the quasi-equilibrium conformation. Supplementary Fig. 7a shows the concentration profile for T4-phage DNA molecules in the $\alpha = 0.45$ nanofunnel at the energy minimum for two field strengths for which residence time measurements were made. As the electric field increases, it forces the molecule towards the nanochannel entrance where DNA compression by the funnel is greater and monomer concentration is therefore higher. Numerical minimization of the effective free energy in Supplementary Equation 19 provides average coordinates, $\overline{x_0}$, $\overline{x_N}$ of the leading and trailing ends and the length $L = \overline{x_N} - \overline{x_0}$ of the DNA molecule, as shown in Fig. 4a (main text) and

Supplementary Fig. 5a. Supplementary Equation 19 can also be used to determine the location and the conformation of the molecule at the effective free energy maximum, $F_{\max}$. At this state, the leading section of the DNA molecule has been pulled into the nanochannel. Supplementary Fig. 7b shows the concentration profiles corresponding to these maximum energy conformations for the same field strengths as the quasi-equilibrium conformations in Supplementary Fig. 7a. At lower electric field strengths, the leading end of the molecule must penetrate deeper into the nanochannel to initiate successful entry of the entire DNA molecule into the nanochannel. The minimum and maximum effective free energy states of the DNA molecule described above correspond to the two balanced force conformations. For the first conformation, where the DNA molecule is wholly within the nanofunnel, the position and conformation of a trapped DNA molecule reflects the position of an effective free energy well (Fig. 2c, main text). For the second conformation, the maximum effective energy corresponds to a molecule in a transition state, from which complete nanochannel entry or recoil back into the nanofunnel are equally probable. The energy difference between these two states is equivalent to the effective free energy barrier to nanochannel entry $\Delta F = F_{\max} - F_{\min}$ (Fig. 2c, main text). Theoretical residence times were then calculated using Equation 1 in the main text.

We note that the monomer concentration profiles shown in Supplementary Fig. 7 appear similar to those of molecules compressed against a nanosphere held by optical tweezers within a nanochannel.[17,18] While the elegant experiments of Khorshid et al. offer another force measurement methodology, there are key differences with our work that should be emphasized. First, our work exclusively explores DNA molecules at quasi-equilibrium whereas the measurements of Khorshid et al. (most importantly, the ones that yield concentration profiles like those of the present work) are performed on transient conformations or steady-state conformations far from equilibrium. Second, the presence of a nanosphere that largely occludes the nanochannel significantly impacts fluid flow in the system of Khorshid et al. Third, while it is our opinion that both methods can yield important

information about near equilibrium and dynamic behavior of large polyelectrolytes, the nanofunnels are more directly applicable to nanofluidic devices for the analysis of biological macromolecules given their ability to lower the threshold electric field needed to induce DNA entry into the nanochannels.

***Thermal fluctuations.*** The experimentally measured thermal fluctuations, $\sigma_{x_0}$ and $\sigma_{x_N}$, and their correlation, $\rho$, can be evaluated theoretically by calculating the minimal work, $R_{\min}$, performed by displacing the molecule from its quasi-equilibrium state. This is the sum of the work, $R_i$ performed by displacing each Kuhn monomer:

$$R_{\min} = \sum_{i=0}^{N} R_i \tag{20}$$

If the $i$-th monomer is displaced from its quasi-equilibrium position $x_i$ by a small distance $\delta x$, the restoring force on this monomer linearly depends on the displacement, $f(x_i + \delta x) \sim \delta x$. The work performed upon shifting the monomer by a small displacement $\delta x_i$ is

$$R_i = -\int_0^{\delta x_i} f(x_i + \delta x) d\delta x \simeq -\frac{1}{2} f(x_i + \delta x_i) \delta x_i \tag{21}$$

For given displacements of chain ends, $\delta x_0$ and $\delta x_N$, we approximate the monomer displacement $\delta x_i = \delta x(x_i)$ by a linear function $\delta x(x)$ of the monomer position $x = x_i$ in the undeformed state:

$$\delta x(x) = \delta x_0 + \frac{x - x_0}{x_N - x_0}(\delta x_N - \delta x_0) = \delta x_0 + (x - x_0)\frac{\delta L}{L} \tag{22}$$

Here $\delta L = \delta x_N - \delta x_0$ is the variation of the molecule's extension along the funnel axis from the equilibrium value $L = x_N - x_0$. Combining Supplementary Equations 20 and 21 and approximating the sum over $i$ by integration over the number of monomers $dN(x)$ in the layer of thickness $dx$,

$$dN(x) = c(x)A(x)\, dx \tag{23}$$

we obtain

$$R_{\min} = -\frac{1}{2}\sum_{i=0}^{N} f(x_i + \delta x_i)\delta x_i \simeq -\frac{1}{2}\int_{x=x_0}^{x_N} f(x + \delta x(x))\delta x(x)\, dN(x) \qquad (24)$$

We calculate the concentration, $c'(x')$, of a displaced DNA molecule, by considering the number of monomers, $dN(x)$, that were displaced from a chain segment with length $dx$ in the quasi-equilibrium conformation to a segment with the same number of monomers $dN(x)$ in the higher effective free energy conformation with length $dx'$:

$$dN(x) = c'(x')A(x')\, dx' = c(x)A(x)\, dx, \qquad x' = x + \delta x(x) \qquad (25)$$

Combining Supplementary Equations 22 and 25 we obtain:

$$(c(x) + \delta c(x))(A(x) + \delta A(x))(L + \delta L) = c(x)A(x)L \qquad (26)$$

where $\delta c(x) = c'(x') - c(x)$ is the density variation produced by the displacement $\delta x$.

The solution of Supplementary Equation 26 for $\delta c(x)$, evaluated up to the second order in $\delta A$ and $\delta L$ is

$$\delta c(x) \simeq c(x)\left[-\frac{\delta A(x)}{A(x)} - \frac{\delta L}{L} + \left(\frac{\delta A(x)}{A(x)}\right)^2 + \left(\frac{\delta L}{L}\right)^2 + \frac{\delta A(x)}{A(x)}\frac{\delta L}{L}\right] \qquad (27)$$

Given the relationship between $A(x)$ and $x$ (Equation 2) we find that

$$\frac{\delta A(x)}{A(x)} \simeq 2\alpha \frac{\delta x(x)}{x} \qquad (28)$$

The segments of a stably trapped DNA molecule are typically wholly inside the funnel (they do not penetrate into the nanochannel) and the contribution of entropic elasticity ($f_{\mathrm{el}}$) is much smaller than that of confinement ($f_{\mathrm{conf}}$) and is excluded from our consideration. In the quasi-equilibrium state, the confinement and osmotic forces acting on the monomers are (Supplementary Equations 4 and 18, respectively):

$$f_{\mathrm{conf}}(x) = P_1 k_{\mathrm{B}} T \frac{c(x)\, b^2}{A^2(x)} \frac{\partial A(x)}{\partial x} \tag{29}$$

$$f_{\mathrm{osm}}(x) = -P_3 k_{\mathrm{B}} T v \frac{dc(x)}{dx}, \tag{30}$$

and the electrohydrodynamic force, $f_{\mathrm{eh}}$, is defined in Supplementary Equation 13. Below we calculate these forces for a DNA molecule with ends displaced by $\delta x_0$ and $\delta x_N$ from their average positions:

$$f_{\mathrm{conf}}\big(x + \delta x(x)\big) = \left[1 - 2\frac{\delta A(x)}{A(x)}\right] f_{\mathrm{conf}}(x) \tag{31}$$

$$f_{\mathrm{osm}}\big(x + \delta x(x)\big) = -P_3 k_{\mathrm{B}} T v \frac{d[c(x) + \delta c(x)]}{(1 + \delta L/L)\, dx} \tag{32}$$

$$f_{\mathrm{eh}}(x + \delta x(x)) = \left[1 - \frac{\delta A(x)}{A(x)} + \frac{\delta c(x)}{c(x)\ln[\xi_{\mathrm{H}}(x)/a]}\right] f_{\mathrm{eh}}(x) \tag{33}$$

Using the condition of quasi-equilibrium,

$$f_{\mathrm{conf}}(x) = -f_{\mathrm{osm}}(x) - f_{\mathrm{eh}}(x) \tag{34}$$

one can exclude the confinement force $f_{\mathrm{conf}}(x)$ from Supplementary Equation 31

$$f_{\mathrm{conf}}\big(x + \delta x(x)\big) = -\left[1 - 2\frac{\delta A(x)}{A(x)}\right][f_{\mathrm{osm}}(x) + f_{\mathrm{eh}}(x)]. \tag{35}$$

Combining Supplementary Equations 22-35 we find the minimal work in the case of a funnel with $\alpha = 0.5$:

$$\begin{aligned} \frac{R_{\min}}{k_{\mathrm{B}} T} \simeq \frac{1}{2} \int_{x_0}^{x_N} \Bigg[ & \left(\frac{\delta x(x)}{x} - \frac{\delta L}{L \ln(\xi_{\mathrm{H}}(x)/a)}\right) \delta x(x)(-f_{\mathrm{eh}}(x)) \\ & - 2\frac{\delta x(x)}{x} \delta x(x) f_{\mathrm{osm}}(x)\Bigg] c(x) A(x)\, dx \\ & + P_3 \frac{v}{2} \int_{x_0}^{x_N} \left[\left(\frac{\delta x(x)}{x}\right)^2 + \frac{\delta x(x)}{x}\frac{\delta L}{L} + \left(\frac{\delta L}{L}\right)^2\right] c^2(x) A(x)\, dx \end{aligned} \tag{36}$$

where upon substituting in the equations for $f_{\mathrm{eh}}$ and $f_{\mathrm{osm}}$ (Supplementary Equations 13 and 30, respectively) we obtain

$$\frac{R_{\min}}{k_{\mathrm{B}}T} \simeq \frac{1}{2}\int_{x_0}^{x_N}\left(\frac{\delta x(x)}{x} - \frac{\delta L}{L\ln(\xi_{\mathrm{H}}(x)/a)}\right)\frac{\delta x(x)}{x}\frac{q_{\mathrm{red}}Ex}{k_{\mathrm{B}}T\ln(\xi_{\mathrm{H}}(x)/a)}c(x)A(x)\,dx$$

$$+ P_3\frac{v}{2}\int_{x_0}^{x_N}\left[\left(\frac{\delta x(x)}{x}\right)^2 - \frac{\delta x(x)}{x}\frac{\delta L}{L} + \left(\frac{\delta L}{L}\right)^2\right]c^2(x)A(x)\,dx \tag{37}$$

Note that only adjustable coefficient $P_3$ enters Supplementary Equation 37 explicitly, while coefficient $P_1$ enters this minimum work implicitly through the quasi-equilibrium concentration profile $c(x)$. Coefficient $P_2$ is omitted from the analysis of stably trapped DNA molecules as $f_{\mathrm{el}}$ is negligible in this case as noted above ($P_2$ does enter into the analysis of nanofunnel residence times, Fig. 3a, main text). Substituting Supplementary Equation 22 and collecting similar terms in powers of $\delta x_0$ and $\delta L$ we get

$$\frac{R_{\min}}{k_{\mathrm{B}}T} = A\left(\frac{\delta x_0}{x_0}\right)^2 + B\frac{\delta x_0}{x_0}\frac{\delta L}{L} + C\left(\frac{\delta L}{L}\right)^2 \tag{38}$$

where coefficients $A, B$, and $C$ are

$$A = \frac{D^2 {x_0}^2}{2x_D}\left[P_3 v\int_{x_0}^{x_N}\frac{c^2(x)}{x}dx + \int_{x_0}^{x_N}\frac{q_{\mathrm{red}}Ex_D}{k_{\mathrm{B}}Tx\ln(\xi_{\mathrm{H}}(x)/a)}c(x)dx\right] \tag{39}$$

$$B = \frac{D^2 x_0}{2x_D}\left[2P_3 v\int_{x_0}^{x_N}\frac{(x-x_0)c^2(x)}{x}dx - P_3 v\int_{x_0}^{x_N}c^2(x)\,dx\right. \tag{40}$$

$$\left. +2\int_{x_0}^{x_N}\frac{q_{\mathrm{red}}Ex_D}{k_{\mathrm{B}}Tx\ln(\xi_{\mathrm{H}}(x)/a)}(x-x_0)c(x)dx\right]$$

$$C = \frac{D^2}{2x_D}\left[P_3 v\int_{x_0}^{x_N}\frac{(x-x_0)c^2(x)}{x}dx + P_3 v\int_{x_0}^{x_N}c^2(x)x_0 dx\right. \tag{41}$$

$$\left. + \int_{x_0}^{x_N}\frac{q_{\mathrm{red}}Ex_D(x-x_0)^2}{k_{\mathrm{B}}Tx\ln(\xi_{\mathrm{H}}(x)/a)}c(x)dx - \int_{x_0}^{x_N}\frac{q_{\mathrm{red}}Ex_D(x-x_0)}{k_{\mathrm{B}}T\ln^2(\xi_{\mathrm{H}}(x)/a)}c(x)\,dx\right]$$

The probability, $P(\delta x_0, \delta x_N)$, of a DNA conformation where the respective ends have deviated by distances $\delta x_0, \delta x_N$ is

$$P(\delta x_0, \delta x_N) = \frac{1}{2\pi\sigma_{x_0}\sigma_{x_N}} \exp\left[-\frac{z}{2(1-\rho^2)}\right], \tag{42}$$

$$z \equiv \frac{(\delta x_0)^2}{\sigma_{x_0}{}^2} - \frac{2\rho\delta x_0 \delta x_N}{\sigma_{x_0}\sigma_{x_N}} + \frac{(\delta x_N)^2}{\sigma_{x_N}{}^2}$$

where

$$\sigma_{x_0} = \frac{x_0}{\sqrt{A - B^2/C}} \tag{43}$$

$$\sigma_{x_N} = \frac{x_0}{\sqrt{C - B^2/A}} \tag{44}$$

and

$$\rho = -B/\sqrt{AC} \tag{45}$$

Supplementary Fig. 8 shows the probability distributions calculated using the above method for two representative trapping field strengths, and its comparison with corresponding experimental results. There is good agreement between the theoretical and experimental distributions despite the approximations in the calculations above. The calculations assume a linear displacement function $\delta x(x)$ in Supplementary Equation 22. Supplementary Equations 39-41 also consider a nanofunnel with $\alpha = 0.5$ to keep the algebra more tractable (experimental measurements were made in a nanofunnel with $\alpha = 0.45$).

***Comparison of theoretical and experimental values.*** Determining the values of the fitting parameters $P_1$, $P_2$, and $P_3$ in Supplementary Equations 19 and 39-41 above that yielded the optimal fit of theoretical predictions to experimental values was achieved using a weighted least squares method. The independent data sets that were included in the analysis were the residence times of λ-phage and

T4-phage DNA molecules in the absence of a nanofunnel and in the $\alpha = 0$, $\alpha = 0.45$, and $\alpha = 0.78$ nanofunnels (8 data sets, Fig 3a); the electric field dependent average positions, $x_0$ and $x_N$, of λ-phage and T4-phage DNA molecules in the $\alpha = 0.45$ nanofunnel (4 data sets, Fig. 4a and Supplementary Fig. 5a); and the electric field-dependent fluctuations, $\sigma_{x_0}$ and $\sigma_{x_N}$, and their correlation, $\rho$, of λ-phage and T4-phage DNA molecules in the $\alpha = 0.45$ nanofunnel (6 data sets, Fig. 4d and Supplementary Fig. 5d). Each data point was weighted by the reciprocal of its variance and the sum of squared residuals was minimized using the steepest descent algorithm. The optimal values of the fitting parameters were found to be $P_1 = 2.3$, $P_2 = 1.1$, and $P_3 = 1.4$. We note again that all three fitting parameters are adjusted to optimize the fits to the residence time data while only $P_1$ and $P_3$ affect the fits to the trapping data.

**Supplementary References**


1. Mannion, J.T., Reccius, C.H., Cross, J.D., Craighead, H.G. Conformational analysis of single DNA molecules undergoing entropically induced motion in nanochannels. *Biophys. J.* **90**, 4538-4545 (2006).

2. Reisner, W. *et al.* Statics and dynamics of single DNA molecules confined in nanochannels. *Phys. Rev. Lett.* **94**, 196101 (2005).

3. The Kuhn length is equivalent to twice the persistence length of a polymer. In the case of double-stranded DNA, the Kuhn length is thus ~100 nm.

4. Stigter, D. Interactions of highly charged colloidal cylinders with applications to double-stranded DNA. *Biopolymers **16***, 1435-1448 (1977).

5. Schaefer, D. W., Joanny, J. F. & Pincus, P. Dynamics of Semiflexible Polymers in Solution. *Macromolecules* **13**, 1280-1289 (1980).

6. The free energy densities are equivalent to the free energy of DNA segments contained in the volume A(x)dx, divided by the volume A(x)dx.

7. Rubinstein, M. & Colby, R. H. *Polymer Physics*. (Oxford University Press, 2003).

8. The correlation length describes the extent of excluded volume interactions. At a length scale of $\xi(x) \approx b/\sqrt{c(x)\, v}$, excluded volume interactions are on the order of thermal energy $k_{\mathrm{B}}T$ and density fluctuations are suppressed. Note that on this length scale different chain sections are overlapping and therefore excluded volume interactions are appropriately described by mean-field methods.

9. Dai, L. & Doyle, P. S. Comparisons of a polymer in confinement versus applied force. *Macromolecules* **46**, 6336-6344 (2013).

10. Dobrynin, A. V. & Rubinstein, M. Theory of polyelectrolytes in solution and at surfaces. *Prog. Polym. Sci.* **30**, 1049-1118 (2005).

11. The hydrodynamic resistances of the device microchannels, nanofunnel, and nanochannel were estimated from the widths, depths, and lengths of these elements and were of the order of $1 \times 10^{12}$ Pa s $m^{-3}$ (microchannels), $1 \times 10^{16}$ Pa s $m^{-3}$ (nanofunnel), and $1 \times 10^{22}$ Pa s $m^{-3}$ (nanochannel). The resistance to flow through a nanofunnel trapped DNA molecule was estimated using the concentration profile of a T4-phage DNA molecule (Supplementary Fig. 7a, blue curve) to calculate the mesh size and assuming flow through a series of parallel tubes with diameter $\xi_{\mathrm{H}}(x)$. Integrating along the nanofunnel's longitudinal axis gave a hydrodynamic resistance of the order of $1 \times 10^{20}$ Pa s $m^{-3}$.

12. Long, D., Viovy, J.-L. & Ajdari, A. Simultaneous action of electric fields and nonelectric forces on a polyelectrolyte: motion and deformation. *Phys. Rev. Lett.* **76**, 3858-3861 (1996).

13. Grosberg, A. Y. & Rabin, Y. DNA capture into a nanopore: interplay of diffusion and hydrodynamics. *J. Chem. Phys.* **133**, 165102 (2010).

14. Rowghanian, P. & Grosberg, A. Y. Two cases of reciprocal relations for electric and hydrodynamic currents: a rigid polymer in a nanochannel and a polyelectrolyte gel. *J. Chem. Phys.* **139**, 024902 (2013).

15. Li, Y. *et al.* Universal behavior of hydrogels confined to narrow capillaries. *Sci. Rep.* **5**, 17017 (2015).

16. Menard, L. D. & Ramsey, J. M. Electrokinetically-driven transport of DNA through focused ion beam milled nanofluidic channels. *Anal. Chem.* **85**, 1146-1153 (2013).

17. Khorshid, A.; Zimny, P.; Tétreault-La Roche, D.; Massarelli, G.; Sakaue, T.; Reisner, W. Dynamic compression of single nanochannel confined DNA via a nanodozer assay. *Phys. Rev. Lett.* **113**, 268104 (2014).

18. Khorshid, A.; Amin, S.; Zhang, Z.; Sakaue, T.; Reisner, W. W. Nonequilibrium dynamics of nanochannel confined DNA. *Macromolecules* **49**, 1933-1940 (2016).
